# Non-medial infectious orbital cellulitis: etiology, causative organisms, radiologic findings, management and complications

**DOI:** 10.1186/s12348-020-00213-3

**Published:** 2020-09-07

**Authors:** Seyed Mohammad Bagher Abtahi, Masoomeh Eghtedari, Shahla Hosseini, Mohammad Shirvani, Atefeh Talebi, Nasrin Masihpoor, Sahar Mohaghegh, Anahid Hamidianjahromi, Mohammad Hosseini

**Affiliations:** 1 Poostchi Eye Research Center, Shiraz University of Medical Sciences, Shiraz, Iran; 2 Biostatistician of Colorectal Research Center, Iran University of Medical Sciences, Tehran, Iran; 3 Infectious Ophthalmologic Research Center, Ahvaz Jundishapur University of Medical Sciences, Ahvaz, Iran; 4 Department of Optometry, School of Rehabilitation, Shahid Beheshti University of Medical sciences and health services, Tehran, Iran; 5 Jahrom University of Medical Sciences, Jahrom, Iran

**Keywords:** Orbital cellulitis; infection, Sinusitis, Subperiosteal abscess, Complication

## Abstract

**Background:**

Orbital cellulitis is an ophthalmic emergency, which is associated with vision-threatening adverse effects. The purpose of this study is investigating etiology, radiologic findings, management and complications of patients with non-medial orbital cellulitis.

**Method:**

A retrospective medical record and radiologic file review of patients with infectious orbital cellulitis was performed to detect all patients with non-medial orbital cellulitis who referred to Khalili hospital from 2016 to 2019. Age, sex, origin of infection, size of collection or abscess, medical or surgical management, microbiology, first and final best-corrected visual acuity, duration of admission, and complications was recorded. Patients divided into two groups; medical management and surgical management groups and all of data compared between in this groups.

**Results:**

Of ninety-six patients with infectious orbital cellulitis, 23 cases (14 male, 9 female) were included. Five patients (21.7%) were managed medically and 18 patients (78.3%) were managed surgically. Patients’ age range was 5–70 years old. Most common location for non-medial cellulitis was superior space (66.7% in surgical and 40% in medical group; *p* = 0.511). In 13 cases of surgical group (72.3%) were detected microorganisms. The mean ± SD of collection volume in medical group were 476.5 ± 290.93 mm^3^ and 2572.94 ± 1075.75 mm^3^ in surgical group (*p* < 0.001). Ten patients in surgical group had compressive optic neuropathy. The mean ± SD of collection volume was 3204.97 ± 879.88 mm^3^ in patient with compressive optic neuropathy and 1280.43 ± 880.68 mm^3^ in patient without compressive optic neuropathy (*P* < 0.001). One case complicated by subdural empyema and another case progressed to necrotizing fasciitis.

**Conclusion:**

Non-medial orbital cellulitis is an uncommon but sight-threatening and life-threatening condition. Timely diagnosis and accurate management reduce morbidity and mortality. Combined surgery for patients with superior or supra-temporal and large non-medial abscess is recommended.

## Introduction

Periorbital Infections based on the involvement of structures and soft tissues, anterior or posterior to the orbital septum anatomically are divided into; preseptal cellulitis (occurs anterior to the septum) and orbital cellulitis (involves tissues posterior to the septum) [1, 2]. Although orbital cellulitis is less common, differentiating it from preseptal cellulitis is very critical and requires careful clinical and radiologic evaluation [1, 3]. Clinical symptoms and signs refer to orbital cavity involvement and orbital cellulitis (post-septal finding) including; proptosis, pain on eye movement, conjunctival chemosis, limitation of ocular motility, decrease visual acuity (VA) and afferent pupillary defect. In patients with post-septal finding who their ophthalmic examinations are difficult and unclear, radiographic imaging is necessary [1, 4]. There are three main sources for orbital infections; (a) secondary extension from periorbital structures (sinusitis, dacrocystitis, dental infection, skin infection and fasciitis, eye lid lesion such as stye), and orbital and ocular adnexal structures (endophthalmitis, dacryoadenitis), (b) direct inoculation of microorganisms following penetrating trauma and surgery, (c) hematogenous dissemination (sepsis and bacteremia, distant infected sites) [4–8]. Secondary extension from para-nasal sinusitis is the most common cause of orbital cellulitis, specifically involving the ethmoid sinus. Medial wall of orbit is the most common location for sub-periosteal abscess (SPA) formation due to thin paper-like bone of ethmoid sinus and loose adherence to periosteum. Inferior orbital wall adjacent to the maxillary sinus is also susceptible to develop SPA. There are no sinuses adjacent to the lateral wall of the orbit. The superior wall adjacent to the frontal sinus is thicker than the medial and inferior walls, however infections might easily spread to the brain [1, 9, 10]. Orbital cellulitis is an ophthalmic emergency, which is associated with sight-threatening adverse effects, such as loss of vision due to compressive optic neuropathy, central retinal artery and vein occlusion [11, 12]. It can lead to life-threatening complications including meningitis, epidural and subdural empyema (SDE), intracranial abscess, sepsis, and cerebral venous thrombosis and death [1, 2, 9, 13, 14]. Timely diagnosis and rapid treatment reduces morbidity and mortality [15, 16]. The purpose of this retrospective study was to evaluate and compare the etiology, radiologic findings, management and complications of patients with non-medial orbital cellulitis and abscess in order to have better understanding of this sight and life threatening condition.

## Methods

This study was approved by the Research Ethics Board of Khalili hospital affiliated with Shiraz University of Medical Sciences, Iran. It was conducted in accordance with the tenets of the Declaration of Helsinki.

We retrospectively reviewed the medical records and radiologic files computed-tomography (CT) scan and/or Magnetic resonance imaging (MRI)) of all patients who were admitted with a diagnosis of orbital cellulitis at Khalili hospital, Shiraz, Iran, from August 2016 to January 2019. Radiologic images were reviewed by an experienced radiologist. The patients with non-infectious orbital cellulitis were excluded. From cases with infections orbital cellulites, the patients with non-medial orbital cellulitis were included in this study. The medical records were evaluated for age, gender, underlying disease, origin of infection, seasonal distribution, laterality, time of symptoms before admission, duration of admission, afferent pupillary reflex on admission, first and final best-corrected visual acuity, recurrence and complications, medical and surgical management, culture and pathology results, imaging study including location and volume of orbital collection or abscess, and lab data including Erythrocyte Sedimentation Rate (ESR) and White blood cell count (WBC). The patients were allocated into two groups of medical management only and medical and surgical management. Orbital collection or abscess volume (mm^3^) was measured with elipsa formula [17]:

$$ \mathrm{Volume}=\pi \times \frac{4}{3}\times \kern0.5em radius\ of\ Height\times radius\ of\ width\times radius\ of\ length $$

The SPSS software version 24 was used for statistical analysis. The results are statistically described as mean ± Standard deviation (SD) in continuous variables. Also, frequency and percentage of categorical variables were reported. Chi-Square and Fisher exact tests were used to evaluate the association between categorical variables. The normality of continuous variables was checked using the Kolmogorov-Smirnov test. In both situation, non-normality and normality cases, the Mann–Whitney U-test and independent *t*-test were used to compare mean of two groups, respectively. Moreover, one-way analysis of variance was performed to compare between more than two means with Tukey’s multiple comparison. Also, Pearson correlation coefficient was calculated for the relationship between two quantitative variables. Level of significance for statistical tests was 0.05.

## Results

All ninety-six available medical records and radiologic files of patients with infectious orbital cellulitis were reviewed and 23 patients met the inclusion criteria (14 male, 9 female). Five patients were managed medically and eighteen patients had undergone surgical management. Patient age range was 5–70 years old with a mean of 21 years in medical management group and mean of 18.75 years in the surgical management group. Of all cases, 16 out of 23 (69%) were ≤ 18 years and 7 (31%) were > 18 years of age. In the medical group, 3 cases were ≥ 9 years and 2 cases < 9 years. In the surgical group, 14 cases were ≥ 9 years and 4 cases < 9 years (*P* = 0.74) (Table 1).

**Table 1:** Laboratory data, radiological finding, and complications of patients in both medical and surgery groups

	Medical group (*n* = 5)	Surgical group (*n* = 18)	*P* value
**Age (year)**			
< 9	2	4	
			0.74
≥9	3	14	
**Gender**			0.67
Male	3 (21.4%)	11 (78.6%)	
Female	2 (22.2%)	7 (77.8%)	
**Location of collection**			
Superior	2 (40%)	12 (66.7%)	
Inferior	3 (60%)	3 (16/7%)	0.511
Supra-temporal	0 (0%)	3 (16/7%)	
**Volume of collection (mm**^**3**^**)**	476.5 ± 290.93	2572.94 ± 1075.75	**< 0.001**
**ESR (mm/hr)**	17.6 ± 3.78	41.55 ± 20.42	**0.007**
**WBC (Cells/μL)**	8540 ± 1625.73	12,933.33 ± 6680.35	0.67
**Compressive optic neuropathy**	0(0%)	10 (100%)	**0.038**
**Recurrence**			
yes	1 (14.3%)	6 (85.7%)	0.51
no	4 (25%)	12 (75%)	
**Duration of hospitalization day**	3.6 ± 0.89	11.89 ± 8.31	**< 0.001**
**Complications**	0	4	0.346
Vision loss	0	1	
Necrotizing fasciitis	0	1	
Subdural empyema	0	1	
Exposure keratitis	0	1	
**Duration of Symptom before admission day**	3.4 ± 1.52	6.11 ± 6.29	0.174

*ESR* Erythrocyte sedimentation rate, *WBC* White blood cell

**Fig. 1:**
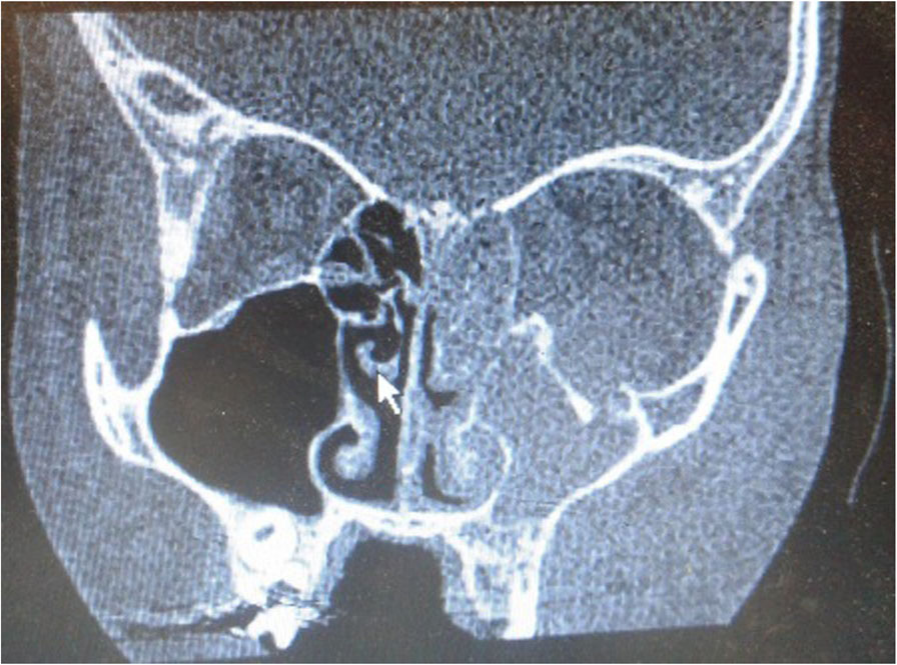
Inferior orbital cellulitis secondary to blow out fracture, Orbital CT scan coronal view revealed inferior wall fracture with total opacity of the left maxillary and ethmoid sinuses

**Fig. 2:**
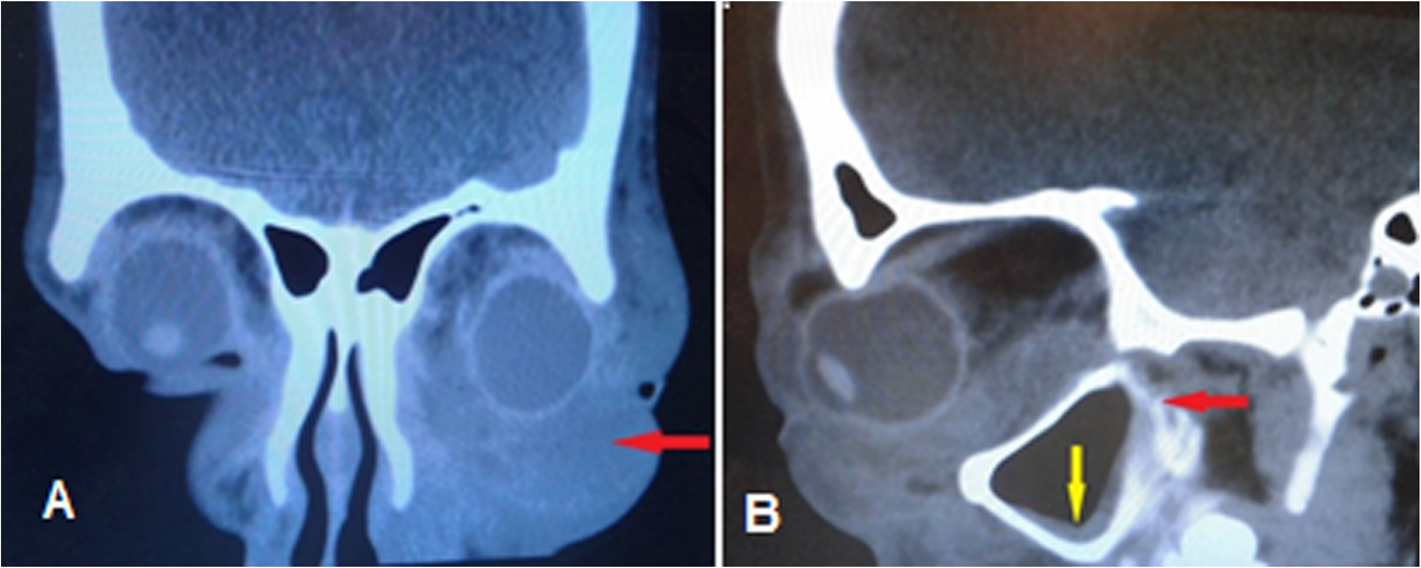
A 19 year-old diabetic patient with inferior orbital cellulitis secondary to dental abscess, **a** Orbital CT scan coronal view revealed inferior orbital abscess (red arrow), **b** axial view revealed mild mucosal thickening of maxillary sinus (yellow arrow) and retro-maxillary opacity (red arrow)

**Fig. 3:**
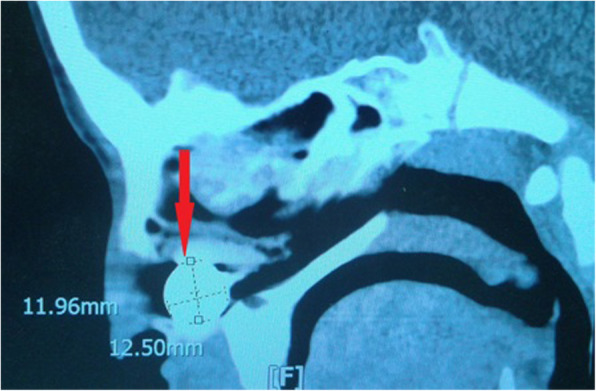
Nasal foreign body (watch battery) lead to orbital cellulitis, Orbital CT scan sagittal view showed a metallic foreign body in the nasal cavity

**Fig. 4:**
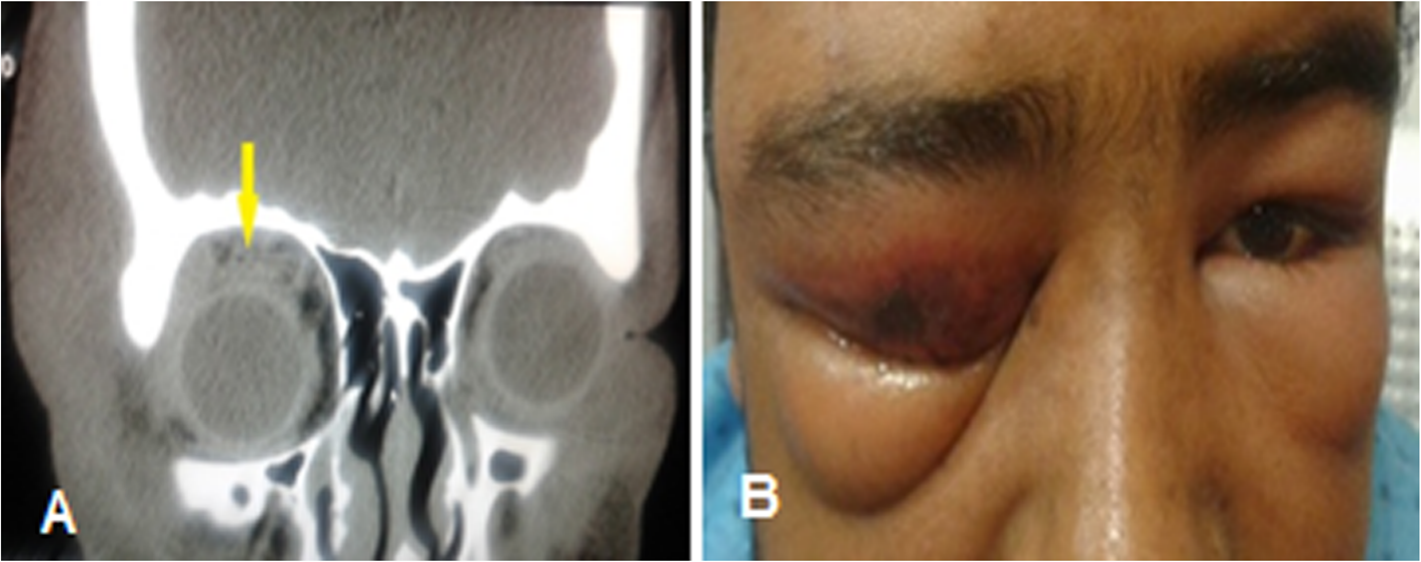
Superior orbital cellulitis due to unknown insect bite that was managed with intravenous antibiotics without any surgical intervention, **a** external eye examination shows eyelids edema and upper lid redness with a small skin necrosis in site of the bite, **b** Orbital CT scan coronal view revealed superior orbital infiltration without abscess formation

Cellulitis or abscess had occurred unilaterally in all patients (13 left-sided, 10 right-sided). Most common predisposing cause was sinusitis (65.2%). Thirteen out of 18 (72.2%) in surgical group and 2 out of 5 (40%) in medical group had sinusitis. Nine patients had pan-sinusitis (*n* = 8 unilateral and *n* = 1 bilateral), 4 patients had fronto-ethmoid sinusitis, and 2 patients had maxillo-ethmoid sinusitis. Other predisposing factors are summarized in the Table 2.Figs. 1, 2, 3, 4

**Table 2:** Summary of the predisposing causes of non-medial orbital cellulitis

Origin of infection	Frequency (%)	Type of management
Sinusitis	15(65.2%)	Medical: 2 (13%) Surgical: 13 (87%)
Super-Imposed infection on chicken pox blisters and herpes zoster vesicles	2(8.7%)	Surgical
Blount trauma with inferior wall fracture (Fig. 1)	1(4.3%)	Surgical
Post lower lid blepharoplasty	1(4.3%)	Medical
Dental abscess (Fig. 2)	1(4.3%)	Surgical
Nasal metallic foreign body (Fig. 3)	1(4.3%)	Medical
Insect bite (Fig. 4)	1(4.3%)	Medical
Unknown	1(4.3%)	Surgical

Orbital cellulitis occurred in winter amongst 8 patients (34.7%), in summer amongst 8 patients (34/7%), in spring amongst 5 patients (21.7%), and in autumn amongst 2 patients (8.7%).

In surgical group, collection or abscess was located in superior of 12 cases (66.7%), inferior of 3 cases (16.7%) and supra-temporal of 3 cases (6.7%), and in medical group, Inferior (Fig. 3) of 3 cases (60%) and superior (Fig. 4) of 2 cases (40%) (*p* = 0.511) (Table 1).

**Fig. 5:**
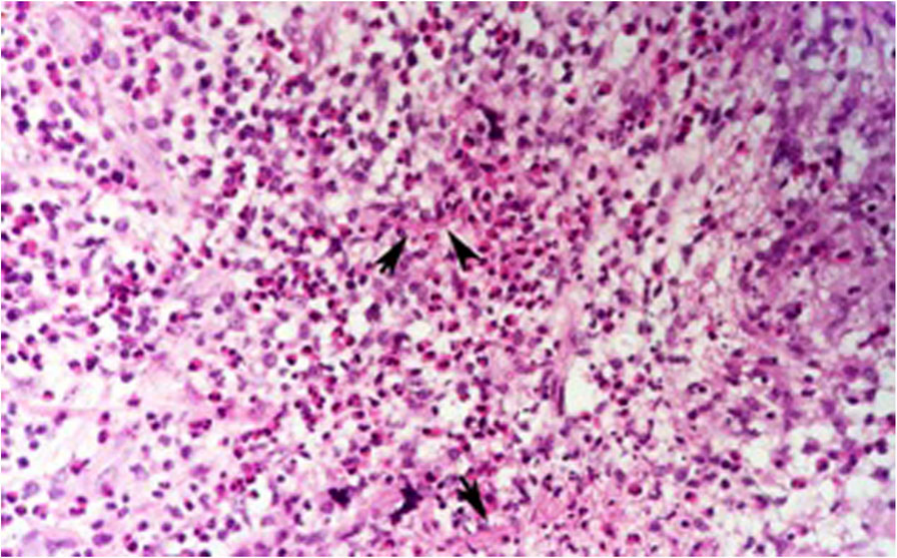
Orbital mucormycosis. Granulomatous inflammation of orbital soft tissue with infiltration of many eosinophils. Note the presence of hyphea (arrows) in the background. Hematoxylin and Eosin × 400

In all cases that underwent surgery, intra-operatively, pus, collection or infected tissues were detected and sent for microbial culture or pathology. Five out of eighteen cases (27.7%) were culture negative, and 13 out of 18 cases (72.3%) culture or pathology were positive. In one case, intra-operatively no pus was detected, but a necrotic mass like lesion was observed, which was sent for pathology and *Mucor* specie*s* was detected (Fig. 5). Common identified organisms were *coagulase-negative staphylococci* (*n* = 3), *Staphylococci aureus* (*n* = 2), *Streptococcus pneumoniae* (n = 2), *Staphylococci epidermidis* (n = 2), *Haemophilus influenzae* (*n* = 1), *Staphylococci haemolyticus* (n = 1), mixed growth (n = 1), and *mucor species* (n = 1), respectively (Table 3).

**Table 3:** Causative microorganism’s results

Organisms	Frequency(***n*** = 13)
*Coagulase-negative staphylococci*	3
*Staphylococci aureus*	2
*Streptococcus pneumonia*	2
*Staphylococci epidermidis*	2
*Haemophilus influenza*	1
*Staphylococci haemolyticus*	1
Mix growth	1
*Mucor species* (Fig. 5)	1

The mean ± SD of duration of symptom before admission in medical group were 3.4 ± 1.52 days and in surgical group were 6.11 ± 6.29 days (*p* = 0.174) (Table 1). The longest duration of symptom before admission was thirty days in *mucormycosis* orbital cellulitis patient.

Duration of hospitalization was longer in the surgical group (mean ± SD = 11.89 ± 8.31 vs 3.6 ± 0.89 days) and was statistically significant (*P* < 0.001). The mean ± SD of hospitalization was 12.67 ± 7.5 days in inferior location, 11.5 ± 15.19 days in supra-temporal location, and 8.93 ± 3.15 days in superior location, which were not statistically significant (*P* = 0.7). Also, the mean ± SD duration of hospitalization was 10.9 ± 4.01 days in patients with compressive optic neuropathy and 9.46 ± 10.36 days in patients without compressive optic neuropathy at the time of admission (*P* = 0.68).

**Fig. 6:**
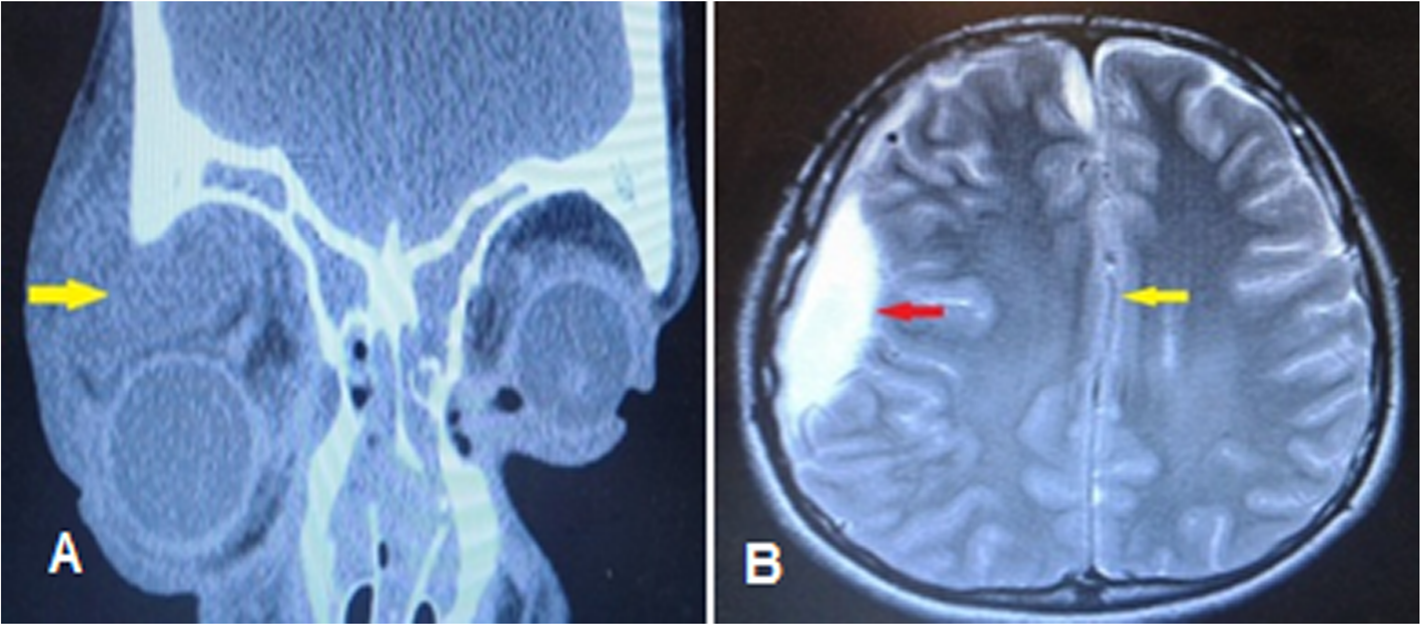
A 10-year-old boy with large supra-temporal orbital abscess progressed to subdural empyema. **a** Orbital computed-tomography scan coronal soft tissue view shows bilateral pansinusitis and right supra-temporal abscess (yellow arrow) with inferior globe displacement. **b** Brain MRI with gadolinium T2 axial view revealed a right-sided subdural empyema (red arrow) with midline shifting to left (yellow arrow)

The mean ± SD of collection volume in medical group was 476.5 ± 290.93 mm^3^ and in surgical group was 2572.94 ± 1075.75 mm^3^ (*p* < 0.001). There was a weak positive correlation between collection volume and the duration of hospitalization, but was not statistically different (Pearson correlation coefficient; r = 0.216 *p* = 0.319). Also, collection volume in supra-temporal (mean ± SD = 3650.36 ± 1041.92 mm^3^) was more than superior (2184.56 ± 1123 mm^3^) and inferior (1193.39 ± 1132.08 mm^3^) locations (*p* = 0.02). The largest abscess was in a 10 year-old boy with supra-temporal location (abscess volume = 4285.44 mm^3^) due to bilateral pansinusitis that developed to SDE (Fig. 6).

**Fig. 7:**
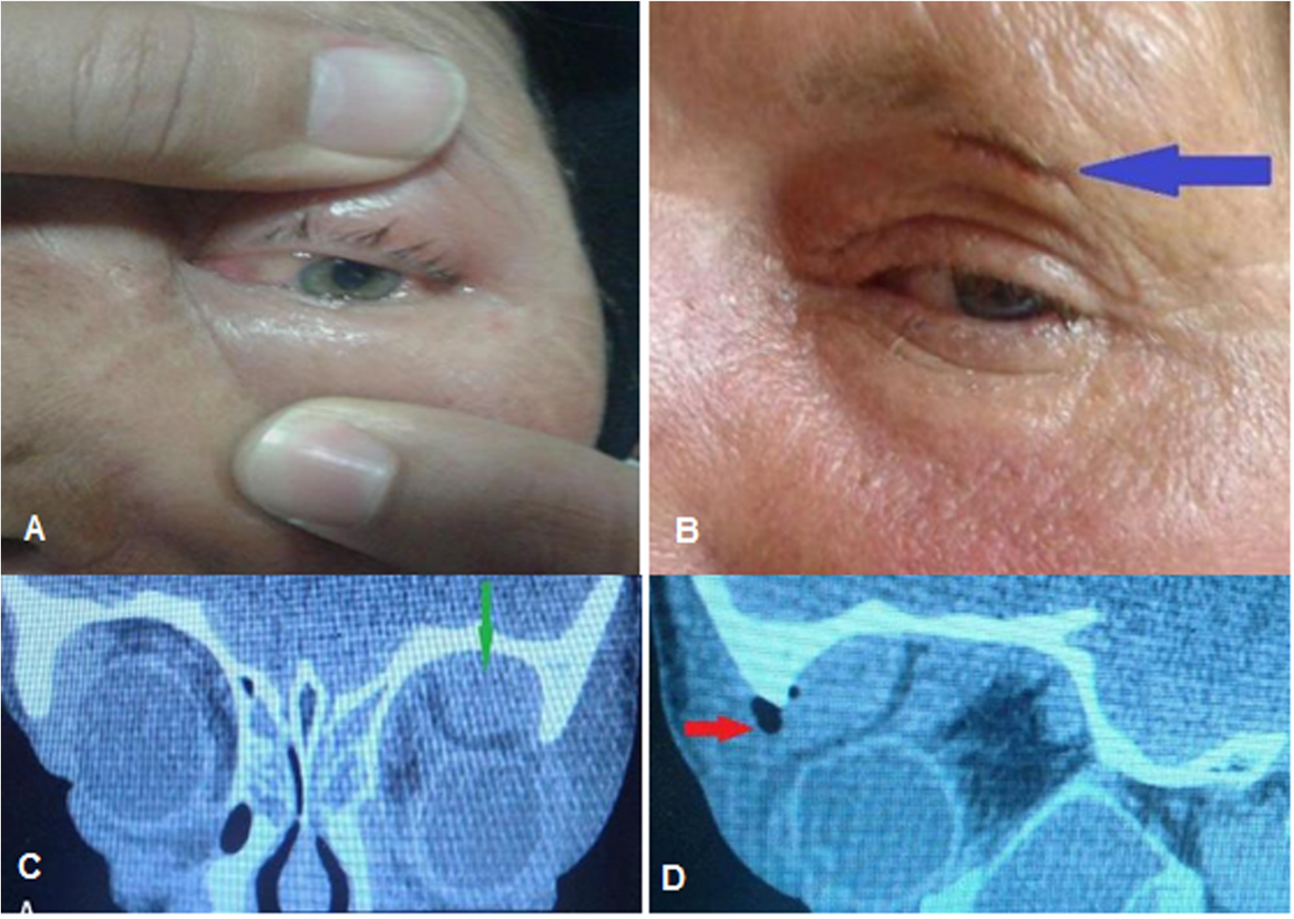
A 62-year-old woman with visual impairment in presenting time, **a** external eye exam shows upper lid swelling and redness with limitation of motion and conjuctival chemosis, **b** 5 day after external abscess drainage with sub-brow incision (blue arrow), **c** orbital CT scan coronal view revealed a left supra-temporal subperiostal abscess (green arrow), **d** and sagittal view showed a superior abscess with hypodense area (red arrow) and compression effect to the globe

There was a statistically significant difference between collection volume and compressive optic neuropathy; the mean ± SD of collection volume was 3204.97 ± 879.88 mm^3^ in patient with compressive optic neuropathy and 1280.43 ± 880.68 mm^3^ in patient without compressive optic neuropathy at the time of admission (*P* < 0.001). There was no patient with compressive optic neuropathy in the medical group, but ten patients in the surgical group had reduced VA with various degrees due to compressive optic neuropathy (*p* = 0.038). Out of the ten patients with VA impairment, nine recovered to normal, and one patient with supra-temporal abscess with volume of 4217.76 mm^3^ and had one episode of recurrence, which ended up with 2/10 snellen VA (Fig. 7).

Although the mean ± SD of ESR was 41.55 ± 20.42 mm/hr. in surgical group and 17.6 ± 3.78 mm/hr. in medical group and had statistically significant difference (*P* = 0.007), the mean ± SD of WBC was 12,933.33 ± 6680.35 cells/μl in surgical group and 8540 ± 1625.73 in medical group, which was not statistically difference (*P* = 0.67) (Table 1).

Common surgical management were combined surgery including external orbital drainage and endoscopic sinus surgery (7 cases of superior collection and all 3 cases of supra-temporal collection) and external orbital drainage (4 cases of superior collection and one case of inferior collection) and endoscopic sinus drainage (2 case of superior collection), respectively.

**Fig. 8:**
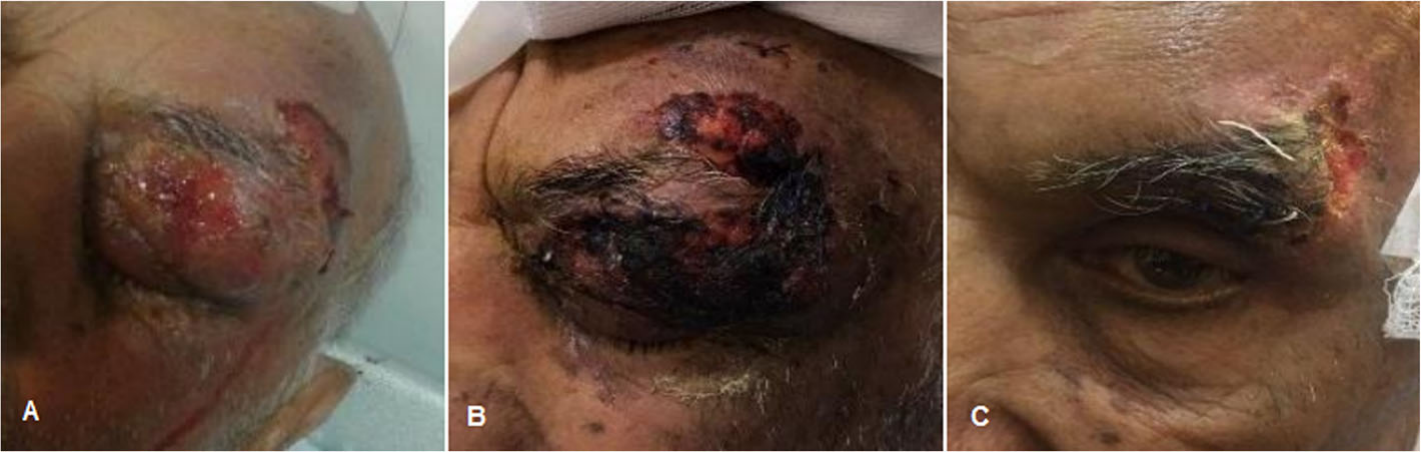
A 70-year-old man with superior orbital cellulitis progressed to necrotizing fasciitis, **a** at presenting time external eye exam shows sever eyelid swelling with ulceration of skin and purulent discharge, **b** 1 day after admission, external eye exam shows multiple area of skin necrosis developing to brow and forehead area, **c** 10 days after minimal surgical debridement and antibiotic therapy complete recovery of eyelid swelling and skin necrosis

Most common antibiotics regimes for the initial empirical therapy were ceftriaxone + clindamycin (87%), ceftriaxone alone (4.3%), clindamycin alone (4.3%), vancomycin + meropenem (4.3%) in one case of orbital cellulitis following dental abscess (Fig. 2). In 5 cases, initial antibiotic therapy due to no responsive to empirical therapy was changed to ceftazidim + vancomycin. In one case, after detecting mucormycosis, antibiotic changed to intravenous Amphotricin-B. In one case, due to progression to necrotizing fasciitis (Fig. 8) antibiotics were change to clindamycin + imipenem + vancomycin and in other patients, and in other patient due to the occurrence of subdural empyema changed to metronidazole + ceftazidime + vancomycin.

Recurrence occurred in one patient in the medical group 5 months after first admission, and during both admission was managed medically with intravenous ceftriaxone, and in six patients in the surgical group (*p* = 0.51). Recurrence in all six cases occurred during hospitalization. Recurrence in one case was managed with change in antibiotic regimes and in the other five cases was managed surgically. In patient with *mucormycosis* orbital cellulitis, two episodes’ recurrence occurred and was managed surgically with trans-conjunctival orbitotomy + debulking of fungal mass. Totally, recurrence occurred in 4 cases with superior collection, 2 cases with inferior collection, and 1 case of supra-temporal collection (*P* = 0.52) (Table 4). Collection volume in patient with recurrence (2328.77 ± 1389.43 mm^3^) was more than the patients without recurrence (2024.62 ± 1295.53 mm^3^), but had not statistically significant (*P* = 0.62).

**Table 4:** Compression of complications, recurrence, duration of admission, and volume of abscess in patients with and without compressive optic neuropathy and in different locations

parameters	Compressive optic neuropathy		*P* value	Location of collection			*P* value
	Positive(*n* = 10)	Negative(n = 13)		Superior (*n* = 14)	Supra-temporal (n = 3)	Inferior (*n* = 6)	
**Complications**							
Yes	3 (75%)	1 (25%)	0.2	2 (50%)	2 (50%)	0 (0%)	0.15
No	7 (36.8%)	12 (63.2%)		12 (63.2%)	1 (5.3%)	6(31.6%)	
**Recurrence**							
Yes	4 (57.1%)	3 (42.9%)	0.245	4 (57.3%)	1 (14.7%)	2(28%)	0.52
No	6 (37.5%)	10 (62.5%)		10 (62.5%)	2 (12.5%)	4(25%)	
**Duration of admission (day)**	10.9 ± 4.012	9.46 ± 10.36	0.68	8.93 ± 3.15	12.67 ± 7.5	11.5 ± 15.19	0.7
**Volume of collection or abscess (mm**^**3**^**)**	3204.97 ± 879.88	1280.43 ± 880.68	**< 0.001**	2184.56 ± 1123	3650.36 ± 1041.92	1193.39 ± 1132.08	**0.02**

Complications including SDE (in case of large supra-temporal orbital abscess) (Fig. 6), vision loss (in case of supra-temporal orbital abscess) (Fig. 2), necrotizing fasciitis (in case of superior orbital abscess) (Fig. 8), and exposure keratopathy (in case of superior orbital abscess) occurred in 4 patients. The mean ± SD of collection volume was 3648.89 ± 887.86 mm^3 in^ patients with complications and 1794.72 ± 1142.24 mm^3^ in patient without complications (*p* = 0.006). Table 5 summarizes the clinical characteristics of patients with complications and Table 6 compares radiologic and laboratory data of patients with and without complication.

**Table 5:** Clinical Characteristics of four non-medial Orbital Cellulitis patients with complications

Complications	Age(M/F)	Source of infection	microbiology	Laboratory data	Underlying disease	Location of involvement	Outcome
**Orbital necrotizing fasciitis**	70(M)	Herpes Zoster Ophthalmicus (HZO)	negative	ESR: 70 mm/hr. WBC:3000 cell/uL	Megaloblastic anemia with pancytopenia	Superior (AV: 2371.81 mm^3^)	Complete recovery
**Subdural empyema**	12(M)	Bilateral pansinusitis	*Streptococci pneumonia*	ESR: 71 mm/hr. WBC:31000 Cell/μL	–	Supra-temporal (AV: 4285.44 mm^3^)	Complete recovery
**Exposure keratitis**	11(F)	Unilateral pansinusitis	*Coagulase-negative staphylococci*	ESR: 50 mm/hr. WBC: 13600 Cell/μL	Known case of cerebral palsy	Superior (AV: 3720.55 mm^3^)	Complete recovery
**Vision loss**	62(F)	Ethmoido-frontal sinusitis	*Staphylocooci aureus*	ESR: 45 mm/hr. WBC: 8200 Cell/μL	–	Supra-temporal (AV: 4217.76 mm^3^)	Permanent vision loss(first BCVA: 2 m CF and final BCVA 2/10)

*M* Male, *F* Female, *ESR* Erythrocyte sedimentation rate, *WBC* White blood cell, *AV* Abscess volume, *BCVA* Best-corrected visual acuity, *CF* Counting fingers

**Table 6:** Compression of volume of collection, ESR, and WBC in patients with and without complication

Parameters	Without complication(***N*** = 20)	With complication(***N*** = 4)	***P***-value
**Volume of collection (mm**^**3**^**)**	1794.72 ± 1142.24	3648.89 ± 887.86	**0.006**
**ESR (mm/hr)**	31.58 ± 18.81	59 ± 13.44	**0.012**
**WBC (cells/uL)**	11,563.16 ± 4607.63	13,950 ± 12,162.65	0.49

*ESR* Erythrocyte sedimentation rate, *WBC* White blood cell

## Discussion

Our study is the first series, reviewing patients with non-medial orbital cellulitis. In our study, five patients with non-medial orbital cellulitis were successfully treated with medical management while many previous studies recommended surgical intervention for patients with non-medial SPAs [18–20]. Oxford and McClay in 2006 established considering new criteria for medical management including; Normal vision and pupil, No ophthalmoplegia, Intraocular pressure < 20 mmHg, Proptosis of 5 mm or less, and abscess width of 4 mm or less on CT scan [21]. In the present series, all ten patients with visual impairment at presentation underwent surgical intervention. Most studies have recommended that any patient at any age who presents or develops visual function impairment must undergo urgent surgical drainage [1, 2, 18]. However, some have recommended indication for surgical drainage including patients ≥9 year old, frontal sinusitis, large SPA (10 mm diameter), unresponsive to initial empirical antibiotic therapy for 48 h, suspicion of anaerobic infection, chronic sinusitis, recurrence of abscess after prior drainage, and infection with dental origin [1, 2, 9, 17, 18]. In our series, 18 patients were underwent surgical intervention.

Orbital cellulitis secondary to dental infection comprises 2–5% of cases and called odontogenic orbital cellulitis. This may arise from any tooth and may involve any location of orbital cavity [22–25]. In a small series of five patients with odontogenic orbital inflammation one patient had preseptal cellulitis and four patients had orbital cellulitis. Medial wall was involved in two cases, inferio-medial in one and inferior wall was involved in another one [23]. In our study, inferior orbital cellulitis occurred in a diabetic patient with poor controlled blood sugar after dental abscess that completely recovered without any complication (first and final Snellen VA were 10/10) after external drainage and dental extraction (Fig. 2). Four pathways were considered for spreading dental infection to the orbital cavity; most common pathway is spreading from roots to maxillary sinus, second pathway is spreading from facial and buccal soft tissues, third pathway is spreading to retromaxillary space and pterygopalatine fossa, and then involvement of orbital cavity via inferior orbital fissure, and fourth pathway is hematogenous seeding from facial and nasal veins [22–27]. It seems that in our case, dental infection had spread to orbital cavity through the second and third pathways.

Recently, some studies noted the collection volume as a new criteria for decision making for medical or surgical management [15, 17, 21]. Michele et al. in a study on 29 pediatric orbital cellulitis reported that abscess volume of less than 1250 mm^3^ does not require surgical management [17]. In our study, the mean ± SD of volume collection were 476.5 ± 290.93 mm^3^ in medical group and 2572.94 ± 1075.75 mm^3^ in surgical group. In our results, larger abscess were associated with dangerous complications (1794.72 ± 1142.24 mm^3^ in non-complicated patients and 3648.89 ± 887.86 mm^3^ in patients with complication *p* = 0.006).

Apparently, Patients with non-medial orbital cellulitis are older than medial orbital cellulitis due to embryology of the sinuses. Ethmoid sinus is present at birth, but other sinuses develop during childhood [1, 9, 21]. In a case series on 24 patients with SPA mean age of patients with medial SPA was 19.45 years and patients with non-medial SPA was 33.25 years [28]. In our study mean age of patients was 19.87 years (18.75 in surgical group and 21 years in medically treated group); therefore, our patients were younger than expected.

In previous series, sinusitis was the most common predisposing causes for orbital cellulitis, and ethmoid sinus was the most common involved sinus, and medial wall of orbit was the most common location for abscess formation [1, 2, 9, 15, 24]. Also, in our study most common origin for non-medial orbital cellulitis was sinusitis (65.2%) and pansinusitis and fronto-ethmoid sinusitis were the common involved sinuses, respectively. In a retrospective study on orbital cellulitis, in antibiotic group 92.6% of patients had medial abscess or collection (medial, supra-medial and infero-medial) and in surgical group 93.3% of patients had medial abscess [15]. Many previous reports have noted that in patients with underlying sinusitis, endoscopic nasal sinus drainage is a useful and appropriate method for medial and infero-medial abscess, but in the case of superior abscess external trans-skin drainage is needed [9, 29–31]. Dewan at el., 2011 in a retrospective study on 24 cases of SPA recommended that cases with large SPA (larger than 2 cm) should undergo combined sinus surgery and external drainage. In this study, 4 patients (16%) had superior SPA and after initial SPA drainage, in 3 of them, re-accumulation occurred [28]. Benjamin at el. in 2015 reported 17 cases of SPA who nine cases had medial SPA, four cases had supramedial SPA and four cases had superior only abscess; in three cases of supramedial SPA that underwent internal sinus surgery only at admission time, re-accumulation occurred that was managed with combined surgery. Also three patients of superior only SPA that underwent combined surgery, successfully treated and another patient was managed with external drainage only. Four cases of medial only SPA were managed medically. They concluded that in cases with superior abscess formation endoscopic ethmoidectomy alone may resulting inadequate drainage lead to re-accumulation of abscess; therefore suggested combined surgery for large medial, superior and combined supramedial SPA [32]. In our study, one case with superior location due to fronto-etmoid sinusitis and one case of inferior collection due to ethmoido-maxillary sinusitis successfully underwent endoscopic sinus drainage. A 5-year-old patient with superior location due to fronto-etmoid sinusitis underwent external drainage with sub-brow incision. Another with superior collection secondary to pansinusitis underwent external drainage, however recurrence episode occurred 3 days later necessitating combined surgery. Other cases with superior and supra-temporal collection secondary to pansinusitis underwent combined surgery. Hence, we suggest combined surgery for patients with superior or supra-temporal collection due to pansinusitis as well as in large non-medial abscesses.

Most common reported organisms from the drained abscess in previous studies are *Staphylococcus aureus*, *Staphylococcus epidermidis*, *Streptococci* species, *Haemophilus influenzae*, *E. coli* and mixed growth including aerobes and anaerobes. Negative culture reported in 25% to 58% of cases [1, 2, 16, 33, 34]. In a retrospective series from four patients with superior SPA, one case was culture-negative and the other, anaerobe rods, B-hemolyticus streptococcus, and poly-microbial were detected [28]. In our study, 13 out of 18 patients (72.2%) that had undergone abscess drainage, were culture-positive, and the most common organism was *coagulase-negative Staphylococcus.*

Permanent or irreversible vision loss is a serious complication of orbital cellulitis that occurs with several mechanisms, and the most common causes is compressive optic neuropathy [1, 2, 26]. In our study, irreversible vision loss occurred in one patients with a large supra-temporal abscess (Fig. 7) who had recollection on the 5th day after initial combined drainage due to compressive optic neuropathy (first Snellen VA 2 m counting finger and defect of pupillary reflex and final Snellen VA 2/10). From 10 patients with initial vision impairment, 9 cases achieved complete vision. After admission and any surgical management, serial ophthalmic examination including VA, pupillary reflex, color vision, eye motility, and amount of proptosis should be evaluated and recorded [1, 2].

In our study, a 70-year-old man with orbital cellulitis secondary to Herpes Zoster Ophthalmicus progressed to upper eyelid necrotizing fasciitis (Fig. 8) Periorbital Necrotising fasciitis (PNF) is a serious and devastating ophthalmic infection associated with mortality and sever visual morbidity. In previous investigations, mortality rate of PNF was reported to be 14.42% and similar to our case, middle area of upper lid was most common involved site [35, 36].

In the present study, a case of primary orbital mucormycosis was presented as inferior orbital cellulitis without any underlying systemic disease and sinuses involvement, but he had a history of swimming in the river 1 week before the eye problems (Fig. 5). Mucormycosis is an uncommon angio-invasive fungal infection associated with immunodeficiency and debilitating diseases such as diabetic mellitus, hematological disorders, and organ transplantation [37, 38]. Rhino-orbito-cerebral mucormycosis (ROCM) is the most common form of mucormycosis and is very rare in immunocompetent patients. Most common involved sinuses in ROCM are ethmoid and maxillary sinuses that may extend to medial and inferior walls of the orbit [37–40]. Mignogna et al. reported six cases of mucormycosis in healthy individuals; five cases had unilateral maxillary sinus involvement that in one of them it was extension to the floor of orbital cavity occurred; and in one case bilateral maxillary and sphenoid sinuses involvement detected [38].

Intracranial complications of orbital cellulitis and para-nasal sinusitis including epidural abscess, SDE, intracranial or cerebral abscess, meningitis, and encephalitis [13]. Microorganisms invade through para-nasal sinuses and orbital cavity to the brain via two pathway; first, direct invasion by the existing micro-perforation or dehiscence and erosion of bony wall, and second; spreading to leptomeninges through communicating valve less veins [13, 41]. Extension of infection through the posterior wall of the frontal sinus is the most common pathway leading to subdural or epidural abscess formation [41, 42]. Nicoli at el. reported six cases of intracranial complications of sinusitis, and all of them had frontal sinus involvement (4 case had pansinusitis, 1 case had large frontal sinusitis, and one case had maxilla-frontal sinusitis) [42]. In our study, a 12-year-old boy with large supra-temporal orbital abscess (abscess volume = 4285.44 mm^3^) secondary to bilateral pansinusutis complicated by SDE during hospitalization was managed by emergency craniotomy without any visual or neurological deficit (Fig. 6). we suggest that any patient with orbital cellulitis; especially in patients with superior orbital cellulitis with frontal sinus involvement presented or developed with neurological sign and symptom should be evaluated for intracranial complications.

Orbital cellulitis secondary to orbital wall fractures is rare and usually occurs in patients with underlying sinusitis. Several mechanisms were postulated for orbital cellulitis secondary to orbital wall fractures like direct extension of microorganisms from infected sinus, impairment of blood supply of orbital cavity, and presence of blood and clots in the sinuses [5, 43, 44]. In present study inferior orbital cellulitis occurred in a 10-year-old boy 1 week after orbital blow out fracture (inferior wall fracture). He had received prophylactic antibiotic that was managed with orbital and sinus fracture repair (Fig. 1). Ben Simon at el. described 4 cases of orbital cellulitis secondary to orbital blow out fracture. All 4 cases had preexisting sinusitis, 3 of them had received prophylactic antibiotics after trauma, however 2 cases progressed to inferior SPA necessitating abscess drainage and while the other two were managed by endoscopic orbital and sinus repair [5]; therefore, in patients with orbital cellulitis secondary to orbital wall fractures with SPA, combined endoscopic and orbital drainage and without SPA, endoscopic sinus repair is recommended.

## Conclusion

Although non-medial orbital cellulitis is less common than medial cellulites but associated with more sight-threatening and life-threatening complications. Most common predisposing factor leading to non-medial orbital cellulitis is sinusitis. In surgical management of non-medial SPA secondary to sinusitis, due to large abscess volume and risk of re-accumulation, combined endoscopic sinus surgery and external orbital drainage is recommended.

## Data Availability

All data are available and the methods are clear.

## Abbreviations

VA: Visual acuity
SPA: Sub-periosteal abscess
SDE: Subdural empyema
CT: Computed-tomography
MRI: Magnetic resonance imaging
BCVA: Best-corrected visual acuity
WBC: White blood cell count
ESR: Erythrocyte Sedimentation Rate
SD: Standard deviation
PNF: Periorbital Necrotising fasciitis
ROCM: Rhino-orbito-cerebral mucormycosis
